# Supplementary material for: Comparative Transcriptomic Analysis of Archival Human Vestibular Schwannoma Tissue from Patients with and without Tinnitus
Source: J Clin Med. 2023 Apr 1;12(7):2642. doi: 10.3390/jcm12072642 (PMC10095534; doi:10.3390/jcm12072642)
Supplement: Supplementary file 1 [file jcm-12-02642-s001.zip › jcm-2283442-supplementary.pdf]

SUPPLEMENTAL MATERIAL *for* "Comparative transcriptomic analysis of archival human vestibular schwannoma tissue from patients with or without tinnitus"

Table S1. Individual demographic and clinical values of the VS study population

ID	Sex	Age (years)	Pre-surgical tumor size (mm)	Tumor side (L/R)	Ipsilateral		Contralateral	
					PTA (dB HL)	WR (%)	PTA (dB HL)	WR (%)
**Without tinnitus (n=9)**								
VS.N1	F	61	12	R	54	32%	32	96%
VS.N2[a]	F	18	28	L	120	0%	6	98%
VS.N3	M	37	30	L	120	0%	10	100%
VS.N4	M	69	40	R	38	6%	5	94%
VS.N5	M	50	25	L	41	22%	11	100%
VS.N6	M	27	47	L	40	88%	10	92%
VS.N7[b]	F	58	27	R	64	80%	10	100%
VS.N8[b]	M	49	36	L	120	0%	58	84%
VS.N9	F	79	30	L	120	0%	35	96%
**With tinnitus (n=10)**								
VS.Y1	F	67	17	R	36	80%	14	100%
VS.Y2[a]	F	20	33	L	100	0%	120	0%
VS.Y3[b]	M	39	21	L	13	92%	9	100%
VS.Y4	M	71	21	L	86	0%	30	90%
VS.Y5	M	57	30	L	81	0%	6	98%
VS.Y6	M	28	25	R	24	96%	21	96%
VS.Y7	F	58	18	L	54	74%	16	92%
VS.Y8	M	43	32	L	29	82%	4	98%
VS.Y9	M	25	29	L	36	12%	8	98%
VS.Y10	M	28	18	L	9	100%	8	98%

Age, sex, tumor size, and presurgical pure tone average (PTA) and word recognition (WR) for the ipsilateral ear of patients with VS included in this study. Cases were stratified by presence (n=10) or absence (n=9) of tinnitus. Abbreviations: dB, decibel; F, female; HL, hearing level; ID, anonymized study identification number; L, left; M, male; R, right. Note: [a] Had neurofibromatosis type 2-related VS. The remainder of patients had sporadic VS. [b] Were excluded from the sequencing experiments due to RNA degradation.
